# Evolution of high-order Tamm plasmon modes with a metal-PhC cavity

**DOI:** 10.1038/s41598-022-19435-7

**Published:** 2022-09-02

**Authors:** Liang Li, Haoyue Hao

**Affiliations:** School of Physics and Optoelectronic Engineering, Shandong University of Technology, Zibo, 255000 China

**Keywords:** Photonic crystals, Nanophotonics and plasmonics

## Abstract

We put forward the concept of high-order Tamm plasmon (TP) modes which are illustrated with a simple metal-Bragg mirror cavity. Results show series orders of TP modes are gradually generated through adjusting the thickness of the cavity, for which traditional TP modes only corresponds to the zero-order modes. The reflectance spectra and electric field distributions are compared to demonstrate the consistency of these series of TP modes. Meanwhile, the excitation intensity of different order TP modes are studied. Results show that the excitation intensity is related directly to the TP mode wavelength, and has no relation to the order number. These results might provide new ideas to the study of TP modes and guide the design and optimization of TP based devices.

## Introduction

Optical Tamm states have been studied for many years, which is initially formed at the interface between two Bragg mirrors^1,2^. Tamm plasmon (TP) modes, generated at the interface of a metal and a Bragg mirror, are known as a special type of optical Tamm state^3–5^. TP modes have narrow band absorption peak in the stop-band of the Bragg mirror, which has great potential in optical sensors^6–9^. TP modes can selectively absorb light with specific wavelengths, which transforms the energy of light into a electromagnetic mode and generates optical field enhancement. Meanwhile, TP modes can be generated with both TM and TE polarized lights without assistance of external structures^10–13^. Based on the above characteristics, TP modes attract attention in many kinds of fields, such as source enhancement in telecom bands^14,15^, confined laser^16–18^, perfect absorption^19–21^, tunable filters^22,23^, and light control^24,25^. Recently, TP modes generated in a metal-photonic crystal (PhC) cavity were demonstrated^26^. Combining with the influence of the top layer on the TP modes^9,27,28^, we put forward the concept of high-order TP modes. In this work, we theoretically studied TP modes with a simple cavity consisted with metal film and Bragg mirror, which also can be called as metal-Bragg mirror cavity. Results show that TP modes have many different orders when we increased the thickness of the cavity while only the zero-order TP modes has been widely studied. The consistency in physical mechanism behind TP modes in different orders is discussed referring to the reflectance spectra and the electric field distribution. These results might greatly expand the study and application of TP modes.

## Structure and methods

**Figure 1:**
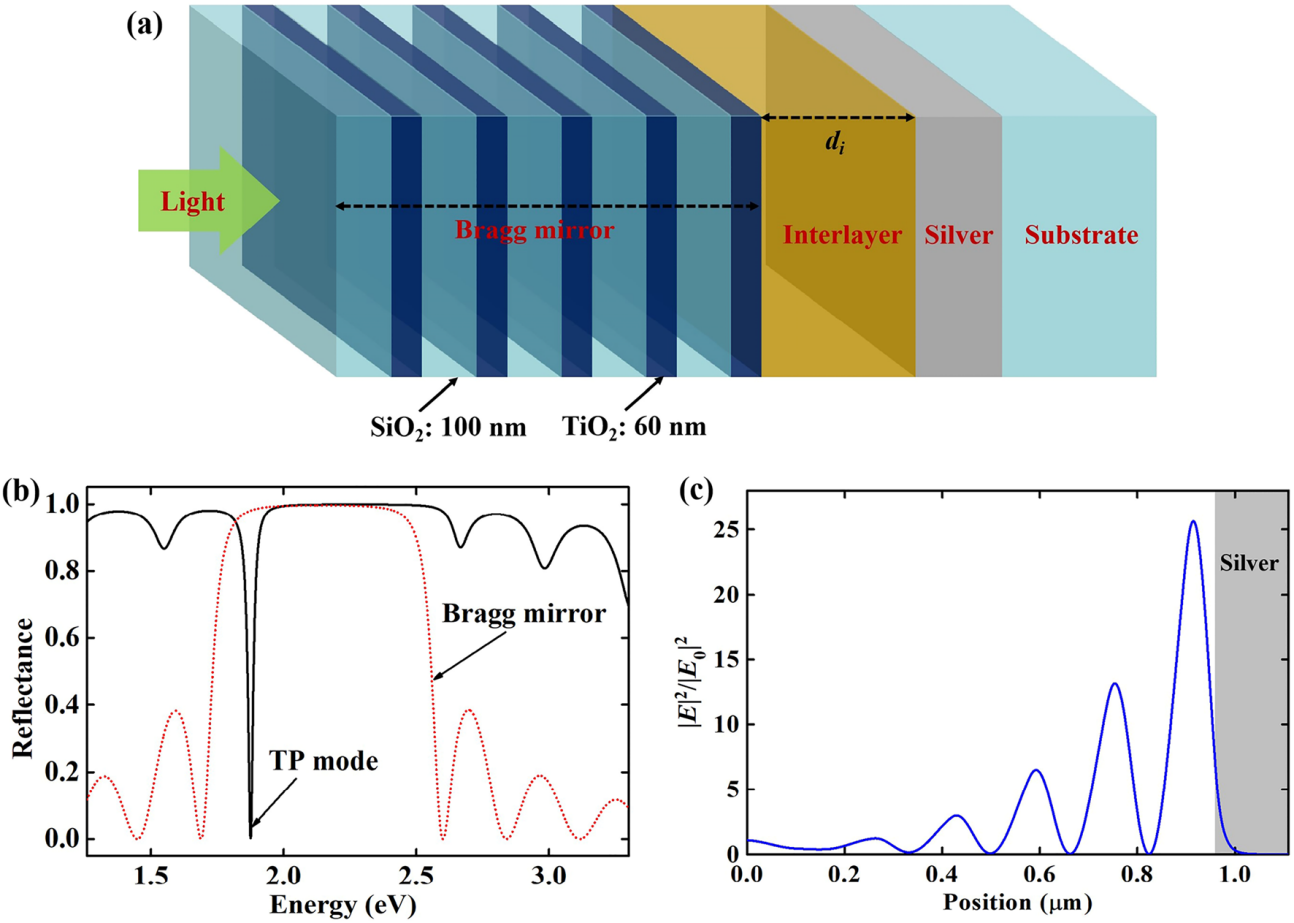
(**a**) Schematic of the extended Tamm structure. (**b**) Reflectance spectrum of the proposed structure. The dotted line is the reflectance spectrum of bare Bragg mirror. (**c**) Electric field distribution of the proposed structure at TP mode.

The extended Tamm structure is a simple cavity consisted with silver film, interlayer and Bragg mirror. To clearly reveal the evolution of TP modes, the structure proposed consists of common materials from previously discussed publications, as shown in Fig. 1a. The alternate dielectric layers of the Bragg mirror are chosen as silicon dioxide (SiO_2_) and titanium dioxide (TiO_2_) with thicknesses of 100 nm and 60 nm, respectively. The thickness of the silver film is set as 150 nm. The permittivity of sliver film can be described by the Lorentz–Drude model^29^. The refractive indices of SiO_2_ and TiO_2_ are set as 1.45 and 2.40. Here, we discuss the preparation process of this proposed structure. First, prepare the silver film and Bragg mirror on silica substrates, respectively. Then, etch a channel on the silver film or the Bragg mirror. Finally, attach the silver film on the Bragg mirror.

The optical transmission properties of the proposed structure are theoretically studied by the transfer matrix approach. Transmission matrix (*T*) and propagation matrix (*P*) are the main matrices in this method, which can be described as^3^

1 $$ T_{k}  = \frac{1}{{t_{k} }}\left[ {\begin{array}{*{20}l}    1 \hfill & {r_{k} } \hfill  \\    {r_{k} } \hfill & 1 \hfill  \\   \end{array} } \right],P_{k}  = \left[ {\begin{array}{*{20}l}    {\exp \left( { - i\phi _{k} } \right)} \hfill & 0 \hfill  \\    0 \hfill & {\exp \left( { - i\phi _{k} } \right)} \hfill  \\   \end{array} } \right]. $$

here, *t*_*k*_ and *r*_*k*_ are the transmission and reflection coefficients of light transmitting from the (*k *− 1)-th layer to the *k*-th layer, which can be derived from Fresnel formula. *φ*_*k*_ is the phase of light propagating in the *k*-th layer. The total transfer matrix of the proposed structure can be deduced as

2 $$ M = T_{1} P_{1} T_{2} P_{2} \cdot \cdot \cdot T_{i} P_{i} T_{s} P_{s} . $$

here, *T*_*i*_ and* P*_*i*_ refer the transmission matrix and propagation matrix for the interlayer. *T*_*s*_ and *P*_*s*_ refer the transmission matrix and propagation matrix for the silver film. The thickness of silver film is thick enough that the transmission light of the proposed structure is approximate to zero. Thus the reflectance and absorptance of the proposed structure can be expressed as *R* = |*M*_21_/*M*_11_|^2^ and *A* = 1 − *R*.

Firstly, the properties of a traditional Tamm structure is investigated through setting the thickness of interlayer at zero, which can be regarded as the traditional Tamm structure. To simplify the discussion, light is vertically incident to the SiO_2_ layer. The period number of Bragg mirror (*N*) is chosen as 6. Figure 1b shows the reflectance spectrum of the traditional Tamm structure. It can be seen that the reflectance spectrum of the traditional Tamm structure has a narrow valley in the stop-band of the Bragg mirror, which is known as TP mode. Figure 1c is the electric field distribution for the traditional Tamm structure at corresponding wavelength of TP mode. It can be found that field enhancement appears near the interface of silver film and Bragg mirror, which reaches ~ 25 times. The main characteristics of TP mode are the narrow valley of reflectance spectrum in the stop-band of Bragg mirror and the electric field enhancement. Thus, under these conditions, the above results demonstrate the traditional Tamm structure without interlayer can excite TP mode.

Initially, researchers have already obtained the excitation condition for TP mode. In this paper, the excitation condition of TP mode can be deduced as^3^

3 $$ r_{BR} r_{S} \exp (2i\phi_{i} ) = 1 $$

here, *r*_*BR*_ is the reflection coefficient of the light incident from the interlayer to the Bragg mirror and *r*_*S*_ is the reflection coefficient of the light incident from the interlayer to the silver film. *i* refers the imaginary unit. *φ*_*i*_ = 2*πn*_*i*_*d*_*i*_/*λ* is the phase of light propagating in the interlayer. We can rewrite Eq. (3) in the form

4 $$ \begin{aligned} & \varphi_{i} + \varphi_{r} = 2m\pi \quad {(}m = 0,1,2 \ldots {)} \\ & \varphi_{r} \approx \pi + \frac{{2n_{i} \omega }}{{\sqrt {\varepsilon_{b} } \omega_{p} }} + \frac{{\pi n_{i} \left( {\omega - \omega_{0} } \right)}}{{\left( {n_{t} - n_{s} } \right)\omega_{0} }} \\ \end{aligned} $$

here, *φ*_*r*_ refers the phase variation induced by the reflection on the silver film and the Bragg mirror. *ε*_*b*_ and *ω*_*p*_ refer the background dielectric constant and plasma frequency of the silver layer. *n*_*i*_, *n*_*t*_ and *n*_*s*_ refer the refractive index of the interlayer, the TiO_2_ layer and the SiO_2_ layer, respectively. *ω*_0_ is the Bragg frequency of the Bragg mirror. In the original report, *m* was limited to 0^3^. From Eq. (4), we can obtain that Tamm mode at a certain wavelength can be excited in series of orders, which corresponds to different *m*. To demonstrate the consistency between different orders of TP modes, we investigate the spectra and the electric field distribution of these TP modes.

## Results and discussion

**Figure 2:**
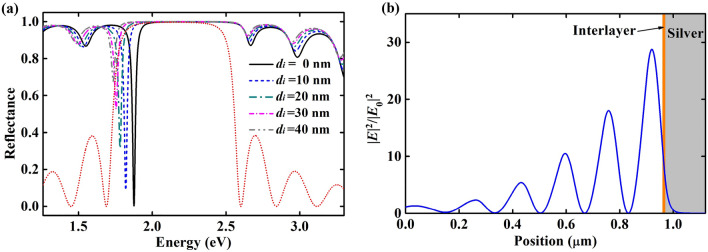
(**a**) Reflectance spectra of the extended Tamm structure at different thickness of interlayer (*d*_*i*_). The dotted line is the reflectance spectrum of bare Bragg mirror. (**b**) Optical field distribution of the extended Tamm structure at 1.8204 eV when *d*_*i*_ = 10 nm.

Firstly, the thickness of the interlayer (*d*_*i*_) is set between 0 and 40 nm, for which *φ*_*i*_ is small enough that *m* = 0. The refractive index of the interlayer (*n*_*i*_) is set as 1.0 (corresponding to air). From Fig. 2a, we can find that TP mode valleys still appear in the reflectance spectra and the valley position red-shifts with increasing *d*_*i*_. TP modes gradually disappear when the TP mode valley shifts out the stop-band of the Bragg mirror (*d*_*i*_ thicker than 40 nm). Figure 2b shows the electric field distribution of the extended Tamm structure at 1.8204 eV (corresponding to the TP mode) when *d*_*i*_ is 10 nm. It can be seen that field enhancement appears in the top layer of Bragg mirror and reaches ~ 30 times. These results demonstrate the generation of TP mode in this extended Tamm structure. That means the TP modes of *m* = 0 (can be called as zero-order) have corresponding optical properties and physical mechanism.

**Figure 3:**
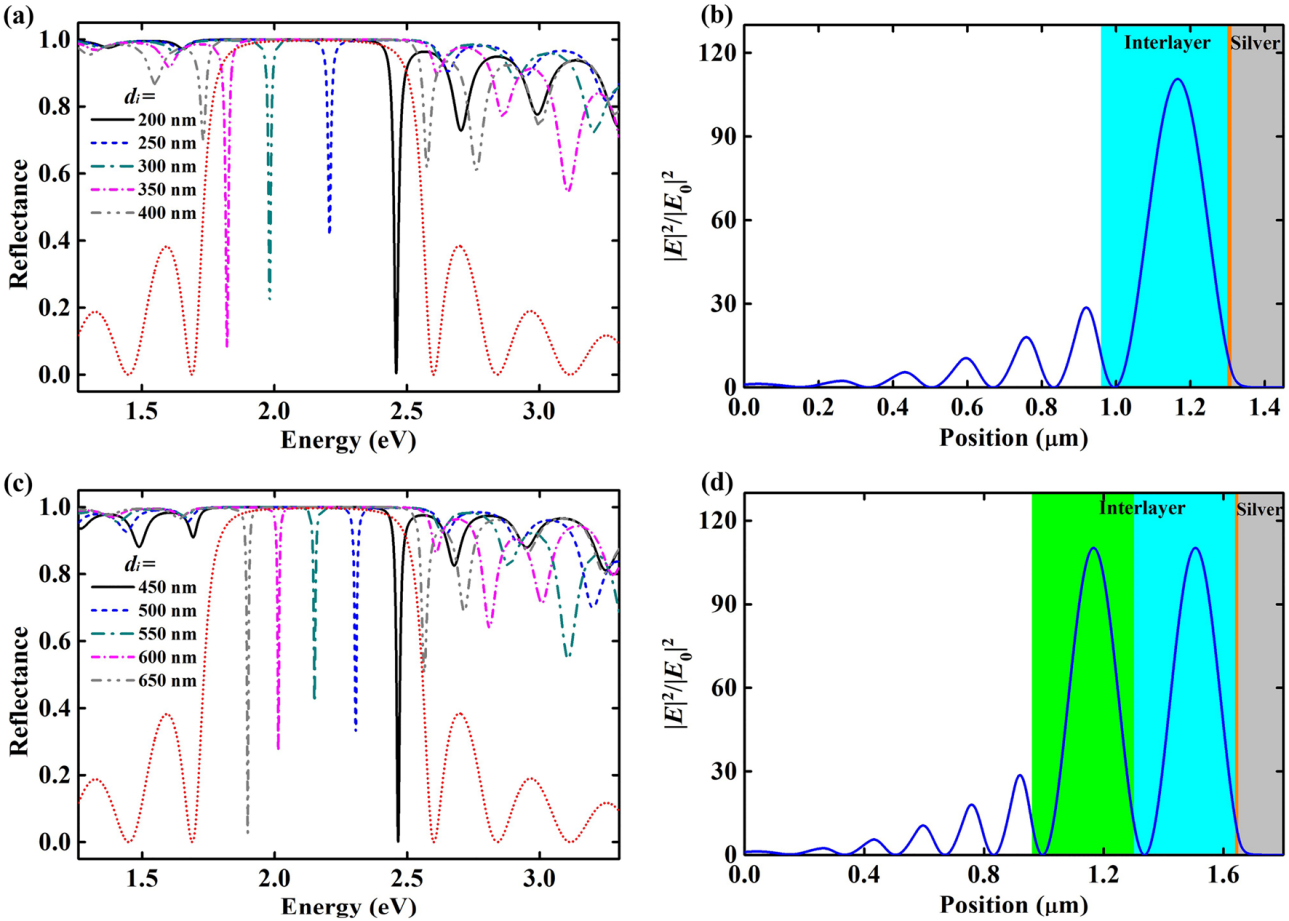
(**a**) Reflectance spectra of the extended Tamm structure at different thickness of interlayer (first-order TP mode). (**b**) Optical field distribution of the extended Tamm structure at 1.8204 eV when *d*_*i*_ = 350.6 nm. (**c**) Reflectance spectra of the extended Tamm structure at different thickness of interlayer (second-order TP mode). (**d**) Optical field distribution of the extended Tamm structure at 1.8204 eV when *d*_*i*_ = 691.2 nm.

As the thickness of interlayer increases, the TP mode valley reappears in the stop-band of Bragg mirror at short wavelength. Figure 3a shows the reflectance spectra of the extended Tamm structure when *d*_*i*_ is 200, 250, 300, 350 and 400 nm. It can be seen that the TP mode valley appears at ~ 2.45 eV when *d*_*i*_ is 200 nm. Meanwhile, the TP mode red-shifts with increasing *d*_*i*_ and gradually disappears again when *d*_*i*_ is thicker than 400 nm. Through estimating the value of *φ*_*i*_, the value of *m* is identified as 1 in this situation. From Fig. 3c, we can find the TP mode valley reappears and disappears again when *d*_*i*_ continue increasing from ~ 450 to ~ 650 nm. Through estimate the value of *φ*_*i*_, the value of *m* is identified as 2 for this situation. Comparing the reflectance spectra of TP modes in different orders, we find both of them have a narrow reflectance valley in the stop-band of the Bragg mirror, which is consistent with the traditional zero-order TP modes.

Moreover, the electric field distributions of different order TP modes are investigated. Figure 3b shows the optical field distribution of the extended Tamm structure at 1.8204 eV when *d*_*i*_ is 350.6 nm, for which *m* = 1. Figure 3d shows the optical field distribution of the extended Tamm structure at 1.8204 eV when *d*_*i*_ is 691.2 nm, for which *m* = 2. The light is chosen at 1.8204 eV (corresponding to 681.2 nm) to compare with *d*_*i*_ = 10 nm. We can easily find that the thickness variation of *d*_*i*_ (*∆d*_*i*_) for adjacent orders is 340.6 nm. It can be deduced that 2*n*_*i*_·*∆d*_*i*_ = *λ.* That means *∆φ*_*i*_ = 1 for adjacent orders at a certain wavelength (equals 681.2 nm for this situation), which matches well with Eq. (4). Comparing the results in Figs. 2b and 3b,d, we can find the electric field distributions in the Bragg mirror, the silver film and the 10 nm thick interlayer close to the silver are basically identical for different orders. The electric field periodically repeats in the other part of the interlayer and the period number is consistent with *m*.

**Figure 4:**
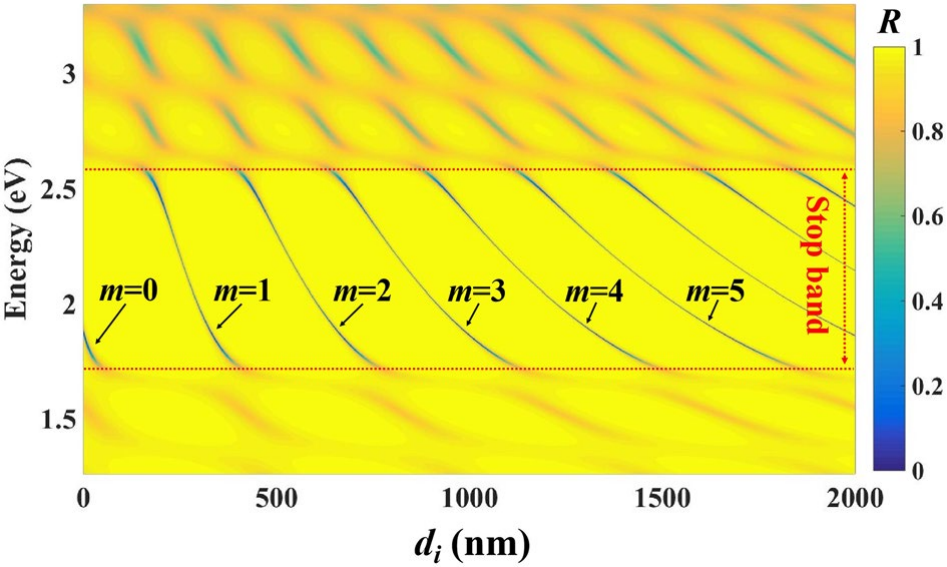
Reflectance spectrum for the proposed structure with different *d*_*i*_.

The evolution of a TP mode as *d*_*i*_ changes from 0 to 2000 nm is shown in Fig. 4. The TP mode valleys in the reflectance spectrum can be found in the stop-band of the Bragg mirror. The valley position regularly changes with the increasing of *d*_*i*_, which forms different series. Corresponding to Eq. (4), each series of valleys refers to an order of TP mode, for which *m* equals 0, 1, 2, 3,… from the left to the right in Fig. 4. Higher order TP modes will appear when *d*_*i*_ increases to suitable thickness. In addition, we can find two or more TP mode valleys appear in the stop band of the Bragg mirror when *d*_*i*_ is thicker than ~ 650 nm. That means the proposed structure can has multi-channels to excite TP modes with suitable thickness of the interlayer and these channels belong to different orders.

**Figure 5:**
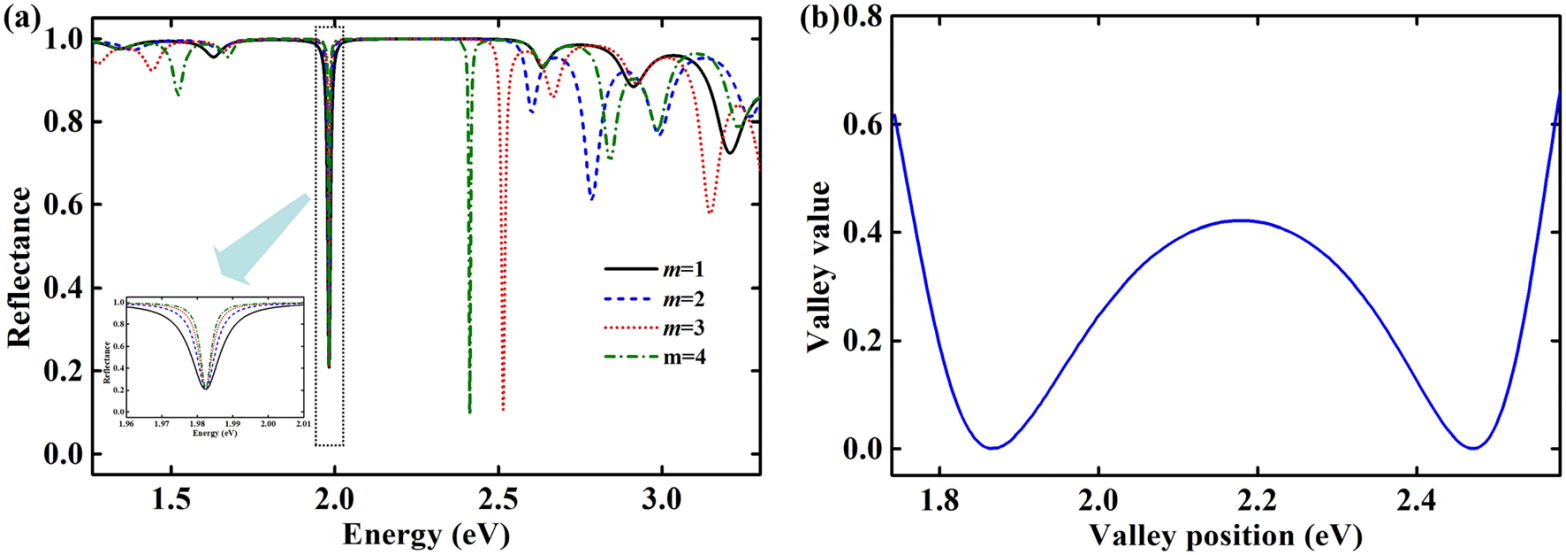
(**a**) Reflectance spectra of TP mode at 1.9824 eV for different orders. The subgraph is the reflectance spectra near 1.9824 eV. (**b**) Dependence of valley value on valley position for TP mode.

The depth of TP valley in the reflectance spectrum is an important property, which can reflect the excitation intensity of TP modes. From Fig. 3a,c, we find the valley value of a TP mode has similar variation trend for first- order and second-order TP modes. Thus the valley value for different orders is investigated at 1.9824 eV (corresponding to a wavelength, λ, of 625.4 nm), as shown in Fig. 5a. The thicknesses of interlayer (*d*_*i*_) for *m* = 1, 2, 3, 4 are 300.0 nm, 612.7 nm, 925.4 nm, 1238.1 nm, respectively. It can be deduced that 2*n*_*i*_·*∆d*_*i*_ = *λ*, which matches well with Eq. (4). Meanwhile, the valley values for different orders are clearly shown in the subgraph of Fig. 5a. It can be seen that the valley value is identical for different orders. That means the excitation intensity of TP modes is related directly to the excitation wavelength. To clarify the excitation rule for TP modes, the relation between valley value and valley position has been investigated, as shown in Fig. 5b. We find that the valley value has two minima distributed at ~ 1.86 eV and ~ 2.47 eV, which nearly reaches zero. Since the absorptance *A* equals 1 − *R*, the proposed structure can realize perfect absorption if a TP mode is generated near ~ 1.86 eV or ~ 2.47 eV. In the middle region of stop-band (Bragg mirror), the proposed structure can generate relatively weak TP modes for the higher valley value. Meanwhile, the valley value dramatically increases when the valley position is close to the boundary of the stop-band (Bragg mirror), which demonstrates TP modes can be excited only in the stop-band of the Bragg mirror.

In addition, it can be found that TP mode valleys become narrower when the order (*m*) increases, as shown in the subgraph of Fig. 5a. The full width at half maximum (FWHM) of the TP mode valley is 0.0098 eV, 0.0062 eV, 0.0040 eV, 0.0034 eV for *m* = 1, 2, 3, 4, respectively. The TP mode valley depends on the excitation condition, as shown in Eq. (4). The phase variation induced by the reflection on the silver film and the Bragg mirror (*φ*_*r*_) remains unchanged when the thickness of interlayer increases. But the light wavelength will have a stronger influence on the phase of light propagating in the interlayer (*φ*_*i*_) when the thickness of interlayer increases. Thus the sum of *φ*_*r*_ and *φ*_*i*_ for higher order TP mode will have more deviation from 2*mπ* when the light wavelength changes. Therefore, the TP mode valleys become narrower when the order (*m*) increases. The structure will have higher sensitivity on the interlayer thickness if the TP mode valley becomes narrower. That means high-order TP modes will have greater potential in optical sensors.

## Conclusion

To summarize, we have investigated TP modes with an extended Tamm structure based on the excitation conditions in the initial work^3^. Through investing the excitation conditions, we find series of TP modes can generate at suitable conditions. All of the different order TP modes have narrow valleys in the reflectance spectra, which is a greatly important property for TP modes. Meanwhile, electric field distributions of different order TP modes are basically identical in the Bragg mirror, the silver film, the 10 nm thick interlayer close to the silver and periodically repeats in the other part of the interlayer. These results show the consistency of the different order TP modes. In addition, the excitation intensity of different order TP modes are investigated with the valley value and FWHM. Results show that high-order TP modes have the same valley value with zero-order TP modes, but has narrower FWHM than zero-order TP modes. That means high-order TP modes will have greater potential in optical sensors.

It is well known that the optical properties of TP structures are dramatically influenced by the nearest layer to the metal film and most applications of TP modes are based on this nearest layer. However, the thickness of this nearest layer is limited for the traditional zero-order TP modes, which highly restricts its potential for large size applications, such as detection of biological tissues and microfluids. Meanwhile, high-order TP modes have more excellent optical properties in the spectrum. The use of high-order TP modes will provide new application fields to TP modes and optimize the design of TP based devices.

## Data Availability

The datasets generated or analyzed during the current study are available from the corresponding authors on reasonable request.
